# Droplet Micro‐Sensor and Detection of Respiratory Droplet Transmission

**DOI:** 10.1002/advs.202401940

**Published:** 2024-06-17

**Authors:** Jiaqi Lu, Xiangdong Chen, Xing Ding, Zhuolin Jia, Mengxiang Li, Mengxi Zhang, Fang Liu, Kun Tang, Xiang Yu, Guoping Li

**Affiliations:** ^1^ School of Information Science and Technology Southwest Jiaotong University Chengdu 611756 China; ^2^ Department of Respiratory and Critical Care Medicine The Third People's Hospital of Chengdu Affiliated Hospital of Southwest Jiaotong University Chengdu 610014 China

**Keywords:** droplet sensors, droplet transmission rules, respiratory droplet detection, wearable devices

## Abstract

Droplet transmission is the primary infection route for respiratory diseases like *COVID‐19* and influenza, but small and low‐cost wearable droplet detection devices are a significant challenge. Herein, a respiratory droplet micro‐sensor based on graphene oxide quantum dots (GOQDs) assembled onto SiO_2_ microspheres by the nebulized natural deposition is presented. Benefiting from the energy dissipation of the microsphere to droplets, the sensor can detect droplets as far as 2 m from coughing. With this sensor, droplet signal variations caused by some factors like distance, speech, angles, and wind directions are explored, and the effectiveness of different protective measures in preventing droplet transmission is evaluated. This droplet detection technology is expected to be utilized for the development of personal detection and protection devices against infectious respiratory diseases.

## Graphical abstract

A micro‐sensor is developed for real‐time respiratory droplet detection. This sensor, featuring a surface structure composed of SiO_2_ microspheres, enhances energy dissipation from droplets and can detect them from distances of up to 2 m. Integrating this sensor into wearable devices can serve as a monitoring tool for susceptible populations.

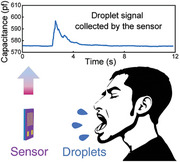

## 1 Introduction

In recent years, the rapid global spread of *COVID‐19* pandemic has brought significant confusion to the formulation of public health policies for respiratory infectious diseases.^[^ ^1^, ^2^, ^3^ ^]^ The World Health Organization (WHO) has clearly pointed out that diseases such as *COVID‐19* and influenza are mainly transmitted through respiratory droplets.^[^ ^4^, ^5^, ^6^ ^]^ Although there have been some classic studies on droplet transmission,^[^ ^7^, ^8^ ^]^ the spread of droplets in the real environment is still vague, so that the social distance varies from 1 to 3 m in different countries.^[^ ^9^, ^10^ ^]^ On the other hand, a fixed social distance fails to reflect individual differences in respiratory droplet transmission,^[^ ^11^ ^]^ thereby inadequately preventing the splash of respiratory droplets on others. Therefore, the respiratory droplet transmission distance of different individuals must be truly reflected through real‐time monitoring devices. Some optical devices, such as high‐speed imaging equipment and the Schlieren technique, have been employed for respiratory droplet observation.^[^ ^12^, ^13^, ^14^, ^15^, ^16^, ^17^ ^]^ For instance, Scharfman and colleagues employed high‐speed cameras to record high‐frame‐rate videos with enhanced contrast through dark‐field setups, providing a visual representation of the complex process from droplet generation in the subject's mouth to droplet fragmentation.^[^ ^12^ ^]^ Simha et al. used the Schlieren technique, which involves optical parabolic mirrors and high‐speed digital cameras, to observe that cough‐generated droplets could travel over 1.5 m.^[^ ^15^ ^]^ Although these devices offer a clear view of droplet transmission, they are prohibitively expensive and cumbersome, generally restricted to laboratory research. This makes them impractical for real‐time personal monitoring and protection against respiratory droplets.

This paper reports a respiratory droplet sensor based on assembling graphene oxide quantum dots (GOQDs) onto silica (SiO_2_) microspheres by the nebulized natural deposition (NND). The sensor's fast response and exceptional sensitivity extend its droplet detection range to 2 m. Some phenomena and laws of respiratory droplet spread have been revealed by the sensor, which not only lays the foundation for the improvement of respiratory epidemic prevention policies, but also provides a new way for personal protection against respiratory droplets.

## 2 Results and Discussion

### 2.1 Fabrication and Characterization of Sensors

**Figure 1:**
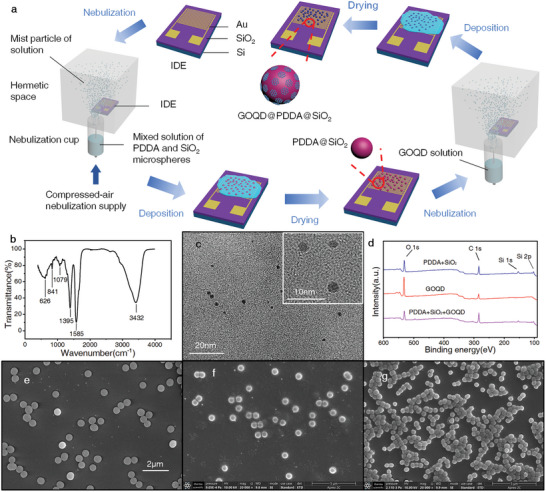
Fabrication steps and characterization of the sensors. a) Process for fabricating sensors by nebulized natural deposition. b) FTIR spectrum of GOQDs. c) TEM and HRTEM characterization results of GOQDs. d) XPS survey spectra of the films in fabrication steps. e) SEM characterization results of pure SiO_2_ microspheres. f) SEM characterization results of the sensor fabricated by nebulized natural deposition. g) SEM characterization results of the sensor fabricated by direct spray deposition.

Given that the main component of droplets is moisture,^[^ ^18^ ^]^ we chose highly hydrophilic GOQDs as the sensitive material for droplet detection and introduced SiO_2_ microspheres to further improve the sensor response.^[^ ^19^, ^20^, ^21^, ^22^, ^23^ ^]^ Meanwhile, the air‐compressed nebulization and self‐assembly method was used to fabricate the sensitive layer for ultra‐fast response of the droplet sensor. Compared with ultrasonic atomizers and spray guns, air‐compressed nebulizers can nebulize the solution into smaller particles (below 5 µm),^[^ ^24^ ^]^ which is beneficial to increase the specific surface area of the sensitive body. However, due to the impact force of the nebulizing airflow, the uniformity of the sensitive film will be deteriorated.^[^ ^25^ ^]^ Therefore, we proposed NND for self‐assembly method to reduce the impact of airflow and improve the uniformity of the sensitive film (**Figure** **1**a). Specifically, a mixed solution composed of poly diallyl dimethyl ammonium chloride (PDDA) and SiO_2_ microsphere (diameter 500 nm) was nebulized upward and deposited naturally on interdigitated electrodes (IDEs). After dying, the GOQD solution (Figure 1b,c) was deposited on the IDEs by NND so that to assemble with PDDA on SiO_2_ microsphere, consisting a GOQD@PDDA@SiO_2_ sensor (called sensor 1). Additionally, a comparison sample (sensor 2) was fabricated using the same method as sensor 1 but without SiO_2_ microsphere.

XPS (X‐ray photoelectron spectroscopy) was employed to analyze the composition of the sensitive films during the fabrication process of the sensor, revealing that the main elements in films PDDA@SiO_2_ and GOQD@PDDA@SiO_2_ were O, C, and Si (Figure 1d). Compared with pure SiO_2_ microspheres (Figure 1e,f) illustrates the successful assembly of sensitive materials onto the surfaces of SiO_2_ microspheres and the substrate of sensor 1. Moreover, the sensitive material distribution on the substrate of sensor 1 (Figure 1e) appeared more dispersed and uniform than that directly sprayed onto the substrate (Figure 1g).

### 2.2 Respiratory Droplet Detection Capability of Sensors

**Figure 2:**
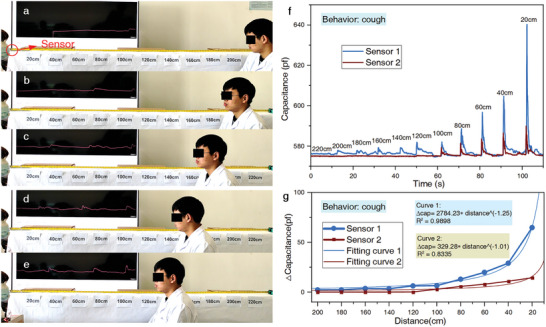
Sensors' capabilities in detecting droplets. a) Experimental scenes for detecting droplets produced by simulated coughs at 200 cm, b) at 180 cm, c) at 160 cm, d) at 140 cm, and e) at 120 cm. f) The droplet signals collected by sensors 1 and 2 from the volunteer coughing during gradual approach. g) Dependence of droplet responses on distances from the volunteer to sensors.

These sensors were employed to monitor droplet emissions from the volunteer at different distances by detecting capacitance changes induced by the moisture content of the respiratory droplets (**Figure** **2**a–e). Figure 2f illustrates the relationship between droplet signals detected by sensors and the distances from the volunteer (Videos S1–S3, Supporting Information). Additionally, signals were collected when the volunteer was moving without coughing, revealing no signal fluctuations during this period (Video S4, Supporting Information). Generally, as the distance between the sensors and the volunteer increased, the droplet responses gradually decreased (Figure 2g). After fitting, it could be found that the relationship between the droplet response detected by the two sensors and the distance followed an exponential distribution, suggesting that the danger of close‐range droplets far outweighs that of distant ones. However, there was a noticeable difference in the response of the two sensors. Sensor 1 exhibited significantly greater droplet responses than sensor 2 at the corresponding distances, whose detection range can reach up to 2 m, far surpassing sensor 2. This implies that the social distancing measures currently adopted by most countries may not provide adequate protection.

### 2.3 Mechanistic Analysis

**Figure 3:**
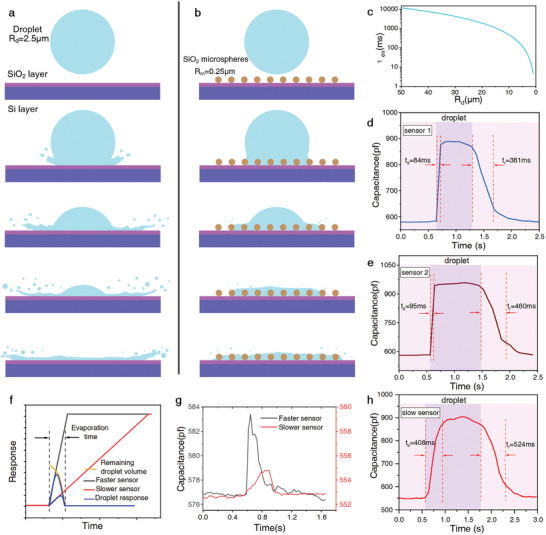
Evaporation of droplets on the sensor surface. a and b) The process of droplets contacting the surface of sensors without SiO_2_ microspheres and with SiO_2_ microspheres, refer to and modify from^[^ ^26^ ^]^ the splashing and spreading of droplets when the droplets hit the contact surface with different roughness taken by the high‐speed camera. c) Evaporation time curves of droplets with different radius (RH = 50%). d) The response/recovery time(t_s_/t_r_) of sensor 1. e) The response/recovery time of sensor 2. f) Schematic diagram of the difference in signal intensity between sensors with different response times when detecting droplets. g) The actual situation of sensors with different response times when detecting droplets. h) The response/recovery time of a slow response sensor, which has a longer response time and the saturated response to droplets is similar to sensor 1.

The results presented above, which show that the droplet detection range of sensor 1 was significantly larger than that of sensor 2, indicated that the microspheres played a crucial role in the droplet detection of the sensor. This may be attributed to the energy dissipation of droplets by microspheres. When a droplet hits a plane, the energy dissipation ϕ, that practically expresses the rate of energy dissipation per unit volume of the fluid, in it can be quantified by the following formula:^[^ ^27^ ^]^

(1) $$\begin{array}{ccc} \phi & = & 2\mu \left[{\left(\frac{\partial u_{r}}{\partial r}\right)}^{2}+{\left(\frac{\partial u_{z}}{\partial z}\right)}^{2}+{\left(\frac{u_{r}}{r}\right)}^{2}+\frac{1}{2}\left({\left(\frac{\partial u_{z}}{\partial r}\right)}^{2}\right.\right.\\ & & \left.\left.+{\left(\frac{\partial u_{r}}{\partial z}\right)}^{2}+2\frac{\partial u_{z}}{\partial r}\frac{\partial u_{r}}{\partial z}\right)\right] \end{array}$$

where μ is the viscosity coefficient of the liquid, *u_z_* and *u_r_* are the velocity components of the droplet in the *z* direction and *r* direction within the cylindrical coordinate system, respectively. According to the simulation calculation of Chamakos et al.,^[^ ^27^ ^]^ it was determined that the total energy dissipation when droplets contact a rough surface is higher compared to that on a smooth surface. This leads to a reduction in droplet splashing,^[^ ^28^ ^]^ and a greater retention of droplets on the sensor substrate (**Figure** **3**a,b), ultimately enhancing the sensor's responsiveness to droplet detection. However, in reality, the distribution of microspheres may not be as regular as depicted in Figure 3b, but their presence enhances the roughness of the sensitive surface, thereby increasing the energy dissipation to droplets. Moreover, variations in microsphere arrangement density led to differences in the sensor's detection range for droplets, with both sparse and dense arrangements affecting the buffering capacity of the sensitive surface against droplets to some extent (Figure S1, Supporting Information).

Meanwhile, respiratory droplets remaining on the sensor substrate are rapidly reduced by evaporation, and the time τ_*ev*_ taken for complete evaporation follows the formula.^[^ ^29^ ^]^

(2) $$\tau_{ev}=\frac{R_{d}^{2}}{\theta \left(1-\mathrm{RH}\right)}$$

where *R_d_* is the droplet radius, θ is the unit of diffusion constant, which is 4.2 × 10^−10^ *m* ^2^ *s* ^−1^ at 25 °C,^[^ ^29^ ^]^ and RH is the relative humidity in the environment. When the relative humidity was 50%RH, the evaporation time was calculated by Equation (2) and plotted in Figure 3c. It could be found that the complete evaporation of small droplets with a radius of 2.5 µm only took 30 ms. However, the response times of sensors 1 and 2 were 84 ms (Figure 3d) and 95 ms (Figure 3e), with mean values of 87.3 and 99.7 ms, respectively (Figure S2, Supporting Information), which were both longer than this evaporation time. This makes it difficult for the sensors to achieve a saturated response so that unsaturated droplet response of sensor has a dependence on its response time (Figure 3f). We conducted an experimental comparison between two different sensors (Figure 3g), sensor 1 and another slower sensor with a response time of ≈400 ms (Figure 3h). Despite their similar saturated response changes (≈300 pF) (Figure 3d–h), the response of the fast sensor was more than twice as large as that of the slow sensor in a cough from the same volunteer at the same distance (Figure 3g). This is another reason why sensor 1 can detect droplets at a significant distance.

### 2.4 Exploration of the Law of Droplet Transmission

**Figure 4:**
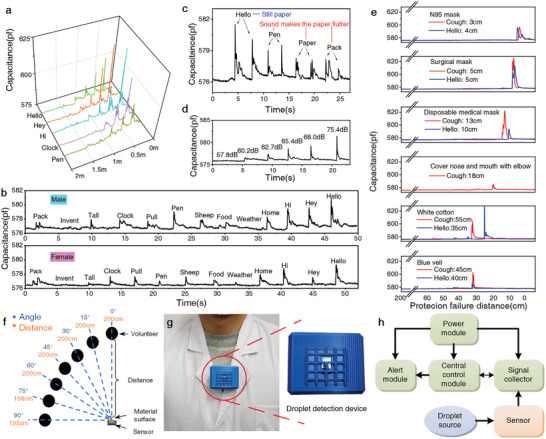
Droplet signals produced during speaking and coughing detected by sensor 1 and droplet detection device. a) Differences in the volunteer's speech droplet signals at different distances. b) The speech droplet signals produced by male and female volunteers at the same distance. c) The fricative and plosive words' speech droplet signals produced by the volunteer at the same distance. d) The speech droplet signals produced by the volunteer saying “Hello” at different volumes from the same distance. e) Different protection failure distances from the sensor to the volunteer in various protective measures while the volunteer speaking and coughing. f) The impact of different angles between sensor 1 and the volunteer on the droplet detection range. g) Photograph of the droplet detection device and its wearing. h) Composition of the droplet detection device.

Using this droplet sensor, we explored the speech droplet spread of some commonly used words (Hello, Hey, Hi, etc.) (**Figure** **4**a). The droplet signals of all tested words could be detected obviously within 100 cm. When the distance is less than 50 cm, speaking these words all produced a large number of droplets (Video S5, Supporting Information). Among them, most words can cause droplet dispersion up to 140 cm away. It is worth noting that the typical greeting words “Hello” can also result in droplets spreading to this distance. Hence, during the epidemic of respiratory infectious diseases, the transmission of speech droplets cannot be disregarded.

For the same distance, we tested the speech droplet responses of male and female volunteers (Figure 4b). It could be seen that gender differences lead to distinct droplet responses by the same word. For most words, the male volunteer produced greater speech droplet responses than the female volunteer (Video S6, Supporting Information). However, some commonalities could also be found among the tested words. The droplet signals of some fricative sounds, especially those words starting with “/h/”, were generally stronger than other words. Among them, “Hello” was prominent. For this reason, we tested the airflow impact and droplet response of “Hello” and some plosive words. When the volunteer spoke a plosive sound word, a strong air flow was ejected from the mouth so that the tissue vibrated visibly, but the droplet response was weak. In contrast, when the volunteer said the word “Hello”, the paper towel only slightly wobbled, but intense droplet response was detected (Figure 4c; Video S7, Supporting Information). This may be because “/h/” as a fricative sound lack vocal reinforcement of the vocal cords, it requires longer intervals to generate sufficient breathing volume for producing sound,^[^ ^30^ ^]^ thus bringing out more droplets. Therefore, during the epidemic, it is not sure if we need consider using other greeting methods instead of saying “Hello”, so as to reduce the droplet transmission to others, which is a difficult choice. Furthermore, the droplet response may also be associated with the volume of the volunteer's speech (Figure 4d; Video S8, Supporting Information). This suggests that whispering in public is indeed necessary.

We also compared the effectiveness of some different protective measures by the droplet sensor. The result in Figure 4e shows that common covers (N95 mask, surgical mask, disposable medical mask, cover nose and mouth with elbow, cotton and veil) can all limit the spread of droplets to a relatively short distance. Among them, the protective effect of N95 masks and surgical masks were the best, which can limit the droplets in 3–5 cm (Video S9, Supporting Information). Therefore, wearing a protective cover will help prevent the long‐distance spread of droplets. However, no matter what kind of shelter it is, it cannot completely block the spread of droplets. This means that even if wearing a mask, you must pay attention to maintaining a safe distance.

Figure 4f shows the influence of the angle between the sensitive surface of the sensor and the volunteer's cough direction on the droplet detection range. It could be seen that changing the angle of the volunteer to the sensor had little effect on the detectable droplet distance. Even though the volunteer was at 90° to the sensitive surface of the sensor, the droplet detection range of the sensor also extended up to 195 cm (Video S10, Supporting Information). This indicates that the sensor possessed a wide detection angle, enabling it to effectively detect droplets across various directions in front of the volunteer.

The real spread distance of droplets may also be affected by the wind directions. For this purpose, we assessed the sensor's droplet detection range under windy conditions. The sensor's droplet detection range differed significantly under conditions of windward (Figure S3a, Supporting Information) and leeward (Figure S3b, Supporting Information), measuring 140 and 220 cm (Figure S4, Supporting Information), respectively. This indicates that the travel distance of the droplets increases further in downwind conditions.

The sensor has been used to develop a wearable droplet detection device (Figure 4g), comprising a droplet sensor, a signal collector, a central control module, an alarm module, and a power module (Figure 4h; Figures S5–S7, Supporting Information). The device had the capability of real‐time monitoring and alarming for droplet splashing, serving as a reminder for individuals to uphold social distancing and mitigate the risk of droplet infection (Video S11, Supporting Information). This holds significant importance for the development of personal droplet protection technology and the improvement of public health strategies.

## 3 Conclusion

We have developed a respiratory droplet micro‐sensor utilizing GOQDs assembled onto SiO_2_ microspheres through NND method. Leveraging the energy dissipation of the microsphere to droplets, this sensor demonstrated remarkable detection capability, reaching distances of up to 2 m from coughing events. Through comprehensive experimentation, we have examined the impact of various factors including distance, speech, angles, and wind directions on droplet signal variations, and assessed the efficacy of different protective measures in mitigating droplet transmission.

The droplet sensor proposed in this paper not only elucidates some previously undisclosed respiratory droplet spread phenomenon but also offers the method for integration into cost‐effective wearable devices, allowing for individual real‐time warning and active protection against respiratory droplets. With the ongoing enhancement of droplet sensors' capabilities, they may potentially be employed to bolster people's ability to combat future respiratory disease outbreaks.

## 4 Experimental Section

### Graphene Oxide Quantum Dots (GOQDs)

The GOQD solution (1 mg mL^−1^, purchased from Nanjing XianFeng‐Nano Co. Ltd., China) was diluted to 0.1 mg mL^−1^ by adding deionized water (DIW), and then sonicated for 30 min to fully disperse.

### Poly Diallyl Dimethyl Ammonium Chloride (PDDA) and Silica (SiO_2_) Microspheres Mixture

PDDA is a strong cationic polymer that can easily capture negatively charged GOQDs in aqueous solution,^[^ ^31^ ^]^ so it was chosen as the assembly substrate of the sensor. Using nano‐SiO_2_ microspheres to support sensitive materials can change the roughness of the sensitive film.^[^ ^32^ ^]^ and improve the sensor's ability to capture droplets.

PDDA solution (20 wt.%, purchased from Aladdin Reagent Co., Ltd., China) was diluted by DIW to 2 mg mL^−1^ and then sonicated for 30 min. SiO_2_ microsphere powder (diameter 500 nm, purchased from Guangyuan Newmate New Materials) was added to DIW, and after ultrasonic treatment for 30 min, a 0.5 mg mL^−1^ SiO_2_ microsphere suspension was obtained. The two solutions were mixed in equal proportions and treated with sonication for 60 min to obtain a mixed solution.

### Interdigitated Electrodes (IDEs)

The structure of the IDEs was fabricated using a silicon wafer as the substrate (7 mm × 11 mm × 0.5 mm), followed by oxidation of the wafer surface to form a layer of SiO_2_ with ≈300 nm thickness. Subsequently, titanium (Ti) was deposited on the silicon dioxide layer via sputtering with a thickness of 100 nm, followed by the deposition of gold (Au) with a thickness of 300 nm using the same sputtering process. The electrode gap spacing was set at 20 µm. Prior to sensor fabrication, the IDEs were cleaned with DIW and then immersed in anhydrous ethanol for ≈5 min.

### Nebulized Natural Deposition

An air‐compressed nebulizer with a spray rate of 0.2 mL min^−1^ was employed to nebulize materials in the liquid cup, generating mist particles. These mist particles were then sprayed upward in a hermetic space so that the mist particles were naturally deposited onto surface of the sensor to form a thin film (Figure 1a).

### Material Characterization

FTIR spectra were obtained with a Fourier transform infrared spectrometer (Nicolet IS10, USA). TEM and HRTEM images were obtained by transmission electron microscope (Talos F200S, USA). XPS spectra were obtained with X‐ray photoelectron spectroscopy (Thermo Scientific K‐Alpha, USA). SEM images were obtained with scanning electron microscope (Thermo Scientific Apreo 2C, USA).

The FTIR spectrum of GOQDs materials shows that there are characteristic peaks near wave numbers 626, 841, 1079, 1395, 1585, and 3432 (Figure 1b), corresponding chemical bonds are C─H, C─O─C, C─O, C─C, C═O, and ─OH,^[^ ^33^ ^]^ respectively.

### Test Methods of Response/Recovery Time

The response time of the droplet sensor is defined as the time it takes for the droplets to come into contact with the sensor surface and reach response saturation. On the other hand, the recovery time is the duration it takes for the sensor to come back to the ambient state from the response‐saturated state. In this study, the response and recovery times are defined based on the time it takes to reach 90% of the saturation value. To assess the response and recovery time of the sensor, the volunteer simulated coughing at a distance of 3 cm from the sensor. The response and recovery time were calculated by the collected droplet signal waveform.

### Informed Consent

The experimental description of human subjects was provided after obtaining informed consent from all volunteers.

## Conflict of Interest

The authors declare no conflict of interest.

## Supporting information

Supporting Information

Supplemental Video 1

Supplemental Video 2

Supplemental Video 3

Supplemental Video 4

Supplemental Video 5

Supplemental Video 6

Supplemental Video 7

Supplemental Video 8

Supplemental Video 9

Supplemental Video 10

Supplemental Video 11

Supplemental Video 12

## Data Availability

The data that support the findings of this study are available from the corresponding author upon reasonable request.
