# Supplementary material for: Droplet Micro‐Sensor and Detection of Respiratory Droplet Transmission
Source: Adv Sci (Weinh). 2024 Jun 17;11(31):2401940. doi: 10.1002/advs.202401940 (PMC11336919; doi:10.1002/advs.202401940)
Supplement: Supplementary file 1 — Supporting Information [file ADVS-11-2401940-s011.pdf]

ADVANCED SCIENCE
Open Access

Supporting Information

for *Adv. Sci.*, DOI 10.1002/advs.202401940

Droplet Micro-Sensor and Detection of Respiratory Droplet Transmission

Jiaqi Lu, Xiangdong Chen, Xing Ding, Zhuolin Jia, Mengxiang Li, Mengxi Zhang, Fang Liu, Kun Tang, Xiang Yu and Guoping Li**

Supporting Information

Droplet Micro-Sensor and Detection of Respiratory Droplet Transmission

Jiaqi Lu, Xiangdong Chen, Xing Ding, Zhuolin Jia, Mengxiang Li, Mengxi Zhang, Fang Liu, Kun Tang, Xiang Yu and Guoping Li**

Table of contents in Supporting Information:

Supplementary text

Supporting figures

Figures S1-S8

Supporting videos

Videos S1-S12

Supplementary text

Fabrication process of the droplet sensor

The process was illustrated in Figure 1a. First, the 2 mg/ml PDDA solution was mixed with the 0.5 mg/ml suspension of SiO_2 microspheres (500 nm in diameter), and 2ml of this mixture was placed into a nebulization cup. Next, the IDEs were positioned with their electrode surface facing upward in a sealed container. The air-compressed nebulizer was then activated to direct the nebulized airflow upwards, transforming the solution into tiny aerosol particles. The nebulization process lasted for 5 minutes, after which the setup was left undisturbed for 15 minutes to allow the aerosol particles to naturally deposit onto the IDEs. Subsequently, the IDEs were left to air-dry, resulting in a surface coated with PDDA@SiO_2. Finally, 2ml of the 0.1 g/ml GOQD solution was assembled onto the previously dried IDEs using the same NND method, resulting in the GOQD@PDDA@SiO_2 droplet sensor.

Selection of the SiO2 microspheres' size and density

To explore the impact of SiO_2 microsphere size on droplet sensor's detection range, solutions of four microsphere sizes with the same concentration (0.5mg/ml) — 200nm, 500nm, 1000nm, and 2000nm (labeled as Size Type1-4 in Figure S1f) — were used to fabricate sensors for comparison (as there are almost no SiO_2 microspheres smaller than 200nm). It was found that variations in microsphere sizes between 200nm and 1000nm had almost no effect on the droplet detection range. However, when the microspheres were too large (2000nm), the detection range decreased. This might be because larger microspheres reduce the surface roughness of the sensor, weakening the energy dissipation of droplets.

The density of microsphere arrangement also impacts the sensor's droplet detection range. This effect was investigated by preparing solutions with varying concentrations of 500nm SiO_2 microspheres (0, 0.05, 0.1, 0.5, 1, 2, 5mg/ml), labeled as Density Type1-7 in Figure S1f, to fabricate sensors with differing density distributions. The sensor without microspheres was sensor 2, while the sensor with 0.5mg/ml concentration was sensor 1, with the distribution shown in Figure 1f. Microsphere distributions for sensors with 0.05, 0.1, 1, 2, and 5mg/ml concentrations were depicted in Figure S1a-e. Figure S1f indicates that overly sparse or dense microsphere arrangements negatively impact the sensor's droplet detection range. The differential box-counting method was used to calculate the fractal dimension (FD) for each microsphere distribution. The FD values from low to high density were 2.0842, 2.0975, 2.1533, 2.252, 2.3487, and 2.403, respectively. A positive correlation was observed between fractal dimension and surface roughness. Based on the droplet detection ranges of these

sensors, it was observed that after reaching a certain fractal dimension, the sensor's ability to detect droplets decreased (Figure S1). This reduction might be due to the critical role of the gaps between microspheres in cushioning droplets.

Supporting figures

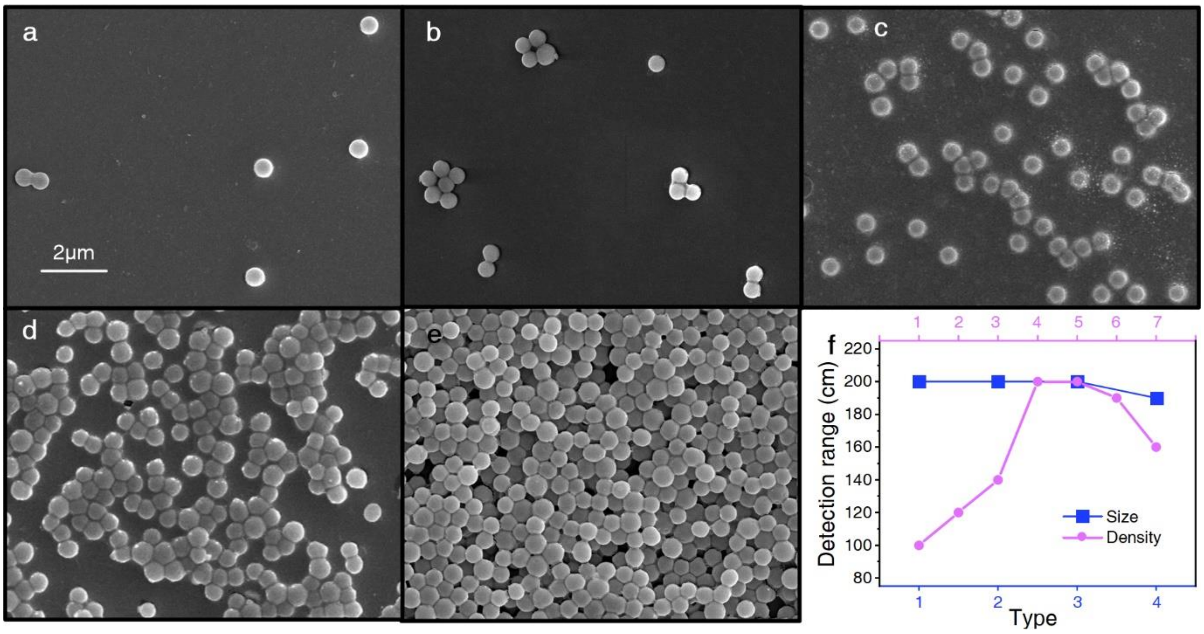

Figure S1. a) Distribution of SiO_2 microspheres at a concentration of 0.05 mg/ml, b) at 0.1 mg/ml, c) at 1 mg/ml, d) at 2 mg/ml, e) at 5 mg/ml, and f) the effects of different microsphere sizes and densities on the sensor's detection range. Size Type 1-4 refer to sensors fabricated with the same concentration (0.5 mg/ml) of microspheres in four different sizes: 200 nm, 500 nm, 1000 nm, and 2000 nm. Density Type 1-7 represent sensors fabricated with 500 nm microspheres at varying concentrations (0, 0.05, 0.1, 0.5, 1, 2, 5 mg/ml) to achieve different microsphere density distributions.

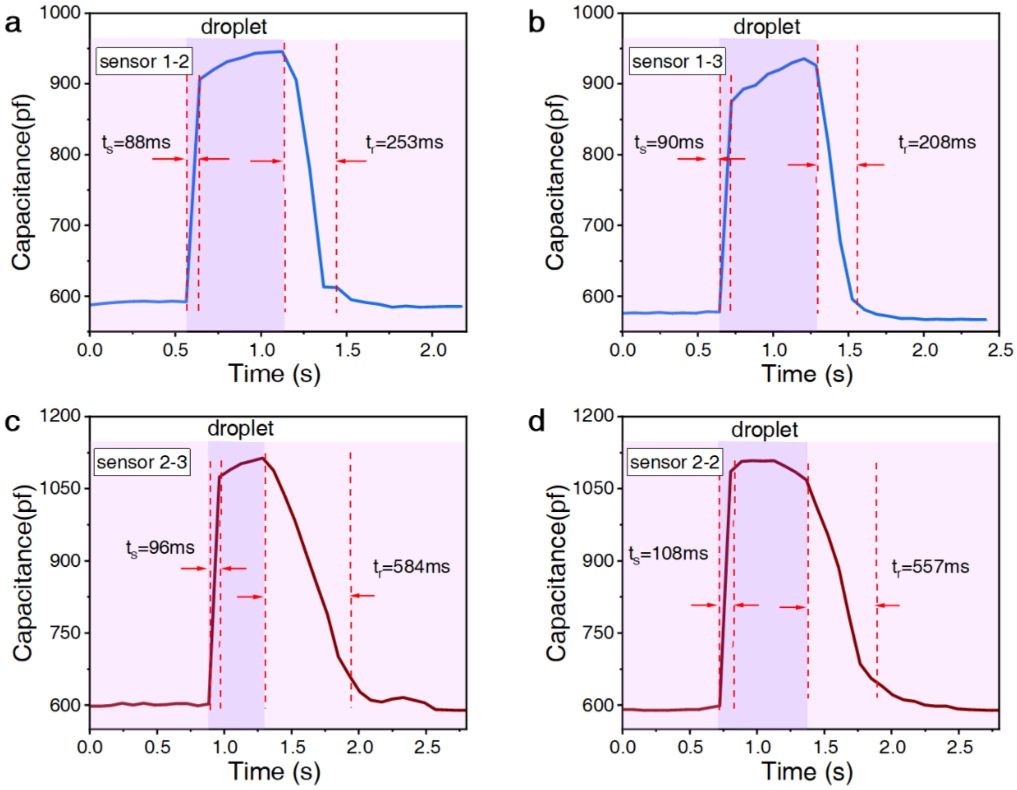

Figure S2. Response/Recovery time for replicated sensor 1 and sensor 2. a) sensor 1-2, b) sensor 1-3, c) sensor 2-2 and d) sensor 2-3.

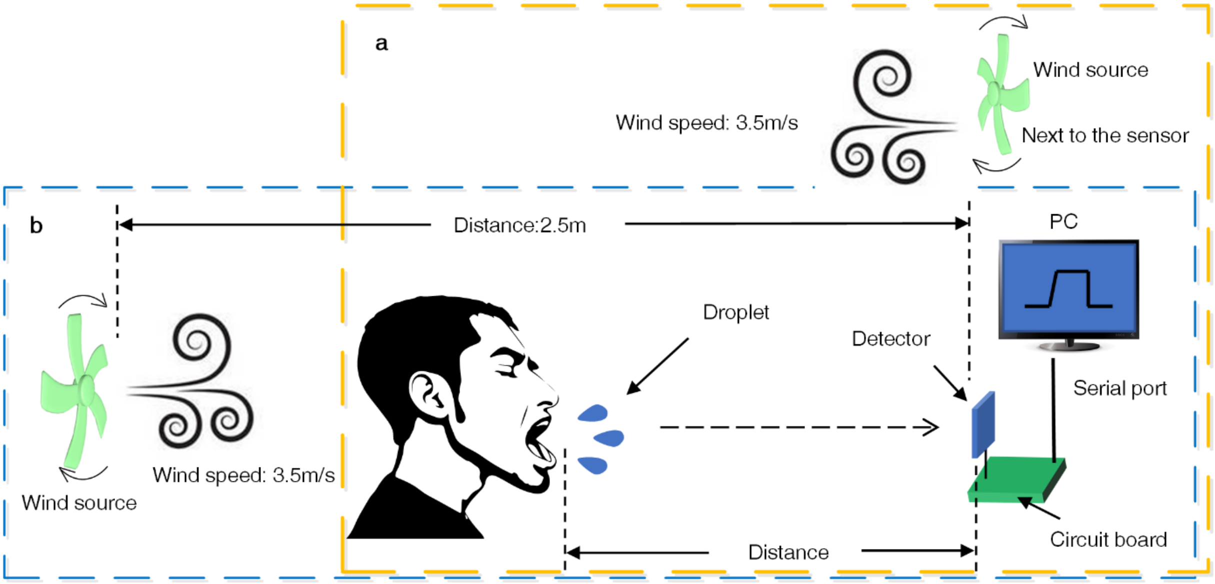

Figure S3. Schematic diagram of wind source placement for detecting droplets under windy conditions. a) Add a wind source next to the sensor, the initial wind speed was about 3.5m/s, blowing towards the volunteer head-on. b) The wind source was behind the volunteer at the distance of 2.5m from the sensor, blowing on the back of the volunteer.

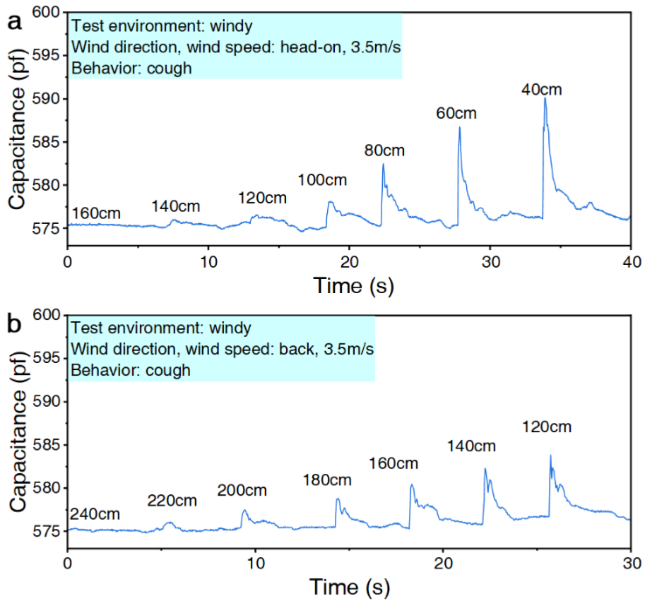

Figure S4. Droplet signals produced by volunteers' coughing at windy conditions, detected by sensor 1. a) The response of droplets at different distances in a head-on blowing environment. b) The response of droplets at different distances in a back blowing environment.

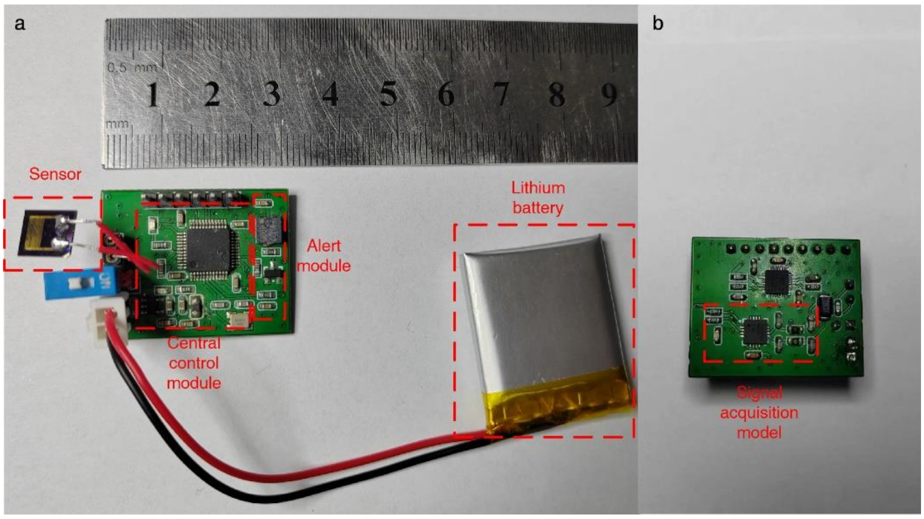

Figure S5. Circuit photos of the droplet detection device. a) Front-Side circuit layout. b) Back-Side layout.

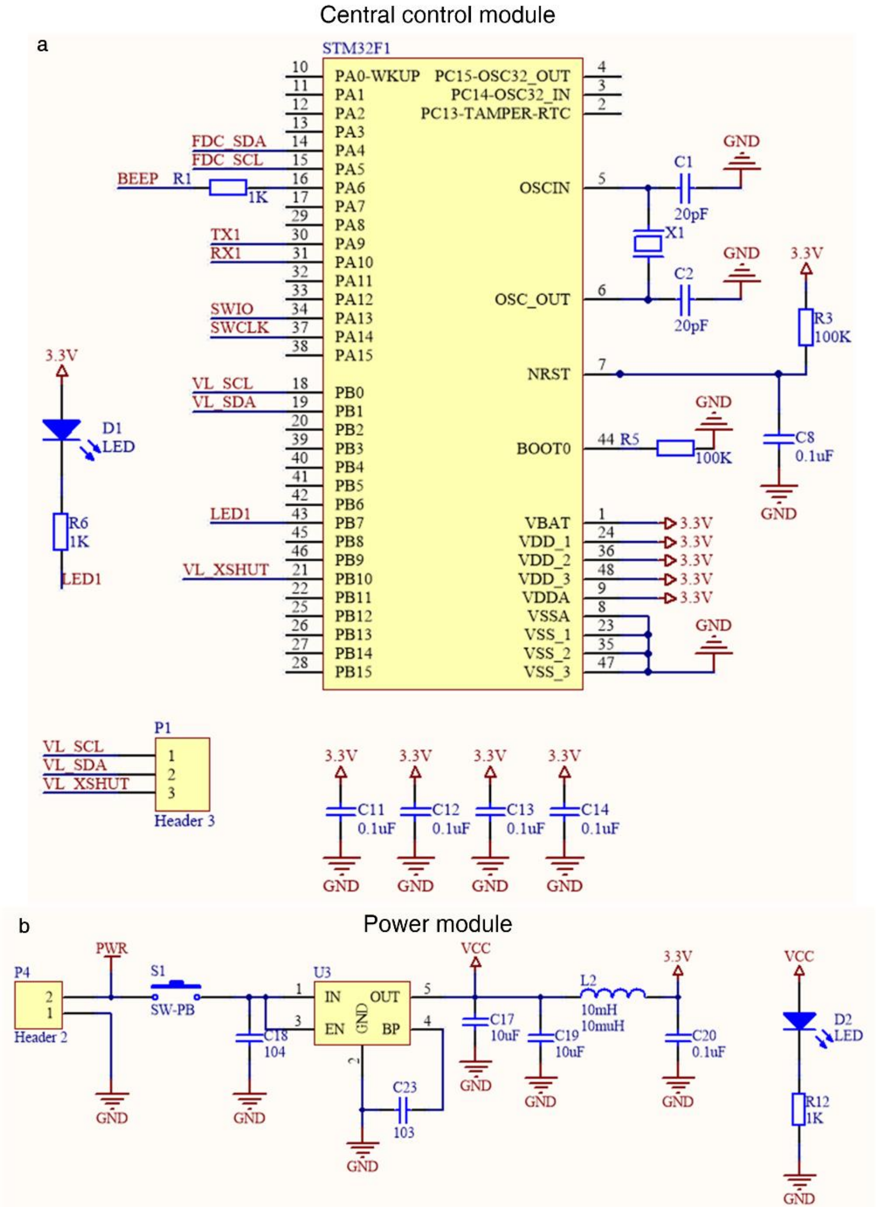

Figure S6. Circuit schematics for a) the central control module, and b) the power module.

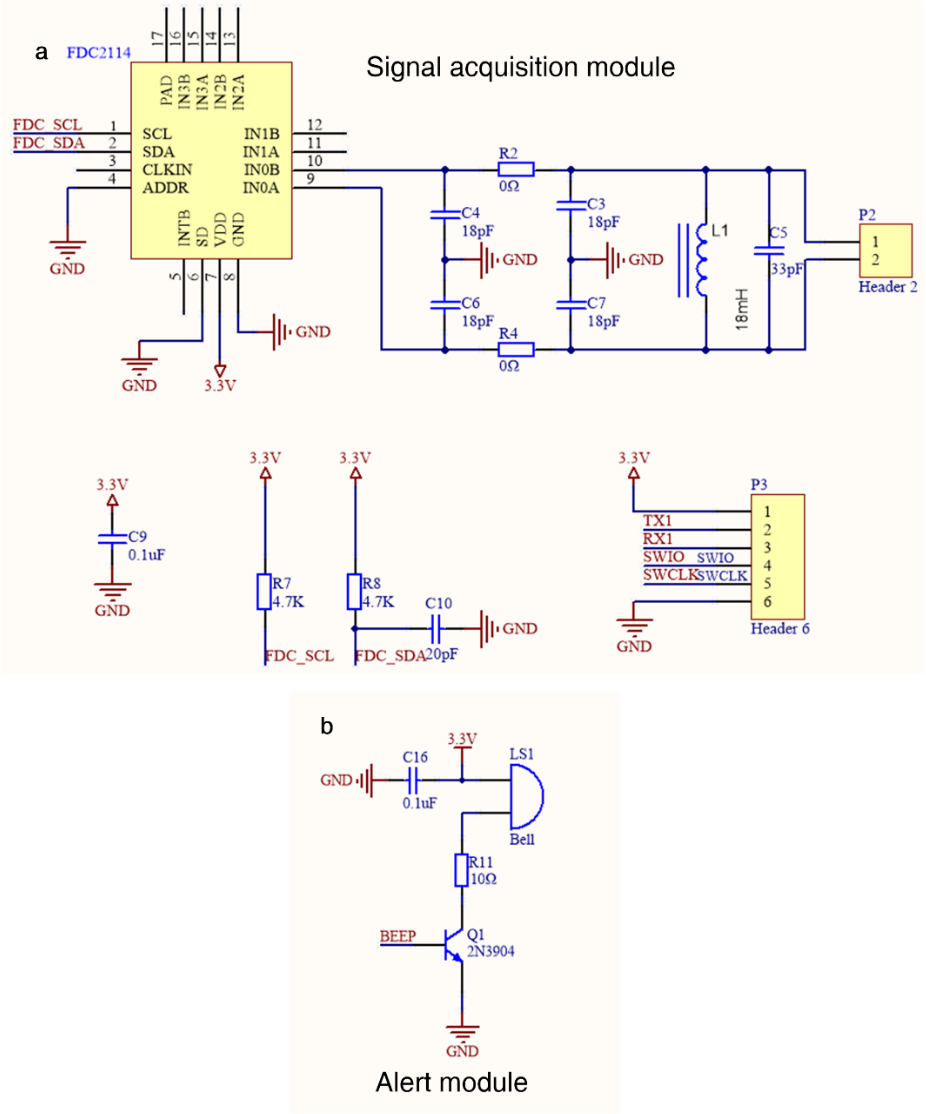

Figure S7. Circuit schematics for a) the signal acquisition module, and b) the alarm module.

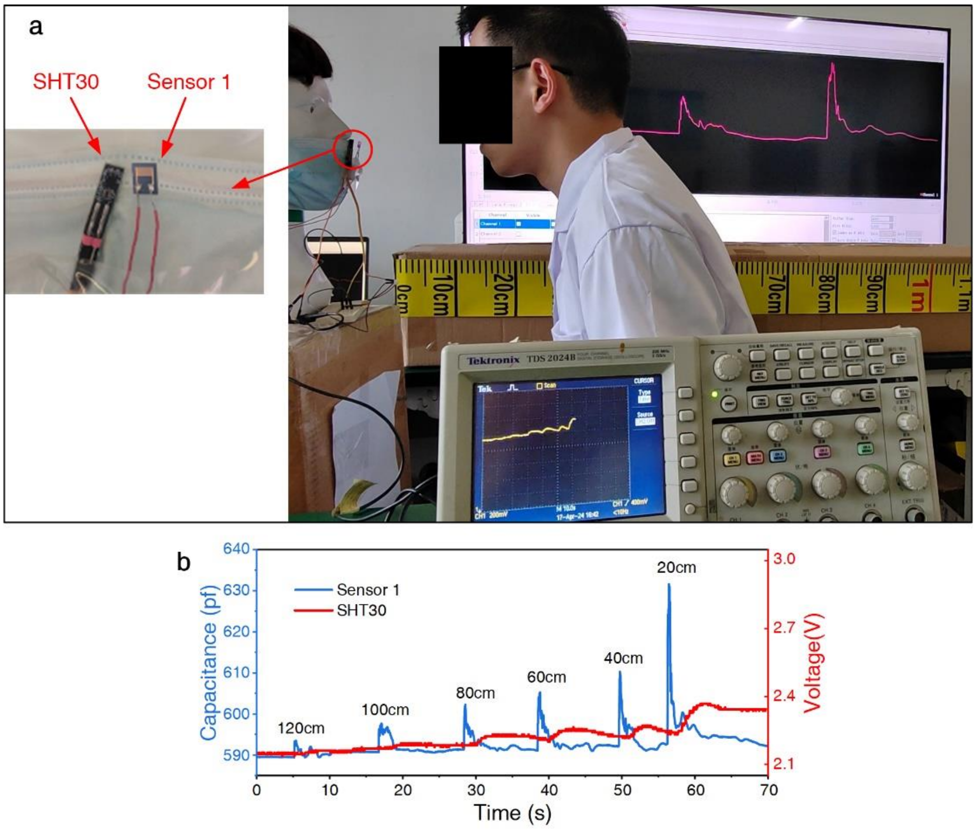

Figure S8. Comparison of droplet detection signals between droplet sensor and humidity sensor. a) Scene for simultaneous collection of droplet signals by sensor 1 and the commercial humidity sensor SHT30. b) Waveform plot of signals from coughing by the volunteer at different distances, as detected by sensor 1 and SHT30.

Supporting videos

Video S1.

Droplet signals from the volunteer coughing at different distances, detected by sensor 1.

Video S2.

Droplet signals from the volunteer coughing at different distances, detected by sensor 2.

Video S3.

Droplet signals from the volunteer coughing at different distances, simultaneously detected by sensor 1 and sensor 2.

Video S4.

Droplet signals from the volunteer moving without coughing, detected by sensor 1.

Video S5.

Droplet signals from the volunteer speaking different words at varying distances, detected by sensor 1.

Video S6.

Droplet signals from the volunteers of different genders speaking different words, detected by sensor 1.

Video S7.

Droplet signals from the volunteer speaking words with fricatives and plosives, detected by sensor 1.

Video S8.

Droplet signals from the volunteer speaking the same word at different volumes, detected by sensor 1.

Video S9.

Droplet signals from the volunteer speaking and coughing while employing various protective measures, detected by sensor 1.

Video S10.

Droplet signals detected by sensor 1 when its sensitive surface is oriented vertically relative to the volunteer.

Video S11.

Alarm demonstration of droplet detection equipment.

Video S12.

Comparison of droplet detection signals between droplet sensor and humidity sensor.
